# Light-dependent regulation of neurotransmitter release from rod photoreceptor ribbon synapses involves an interplay of Complexin 4 and Transducin with the SNARE complex

**DOI:** 10.3389/fnmol.2024.1308466

**Published:** 2024-02-28

**Authors:** Uwe Thorsten Lux, Jutta Meyer, Olaf Jahn, Adam Davison, Norbert Babai, Andreas Gießl, Anna Wartenberg, Heinrich Sticht, Nils Brose, Kerstin Reim, Johann Helmut Brandstätter

**Affiliations:** ^1^Animal Physiology/Neurobiology, Department of Biology, Friedrich-Alexander-Universität Erlangen-Nürnberg, Erlangen, Germany; ^2^Department of Molecular Neurobiology, Max Planck Institute for Multidisciplinary Sciences, Göttingen, Germany; ^3^Neuroproteomics Group, Department of Molecular Neurobiology, Max Planck Institute for Multidisciplinary Sciences, Göttingen, Germany; ^4^Department of Psychiatry and Psychotherapy, University Medical Center Göttingen, Georg-August-University, Göttingen, Germany; ^5^Department of Ophthalmology, University Hospital Erlangen, Friedrich-Alexander-Universität Erlangen-Nürnberg, Erlangen, Germany; ^6^Division of Bioinformatics, Institute for Biochemistry, Friedrich-Alexander-Universität Erlangen-Nürnberg, Erlangen, Germany

**Keywords:** mouse retina, G protein, ribbon synapses, neurotransmitter release, rod photoreceptor, light adaptation

## Abstract

Adaptation of photoreceptor sensitivity to varying light intensities is a fundamental requirement for retinal function and vision. Adaptive mechanisms in signal transduction are well described, but little is known about the mechanisms that adapt the photoreceptor synapse to changing light intensities. The SNARE complex regulators Complexin 3 and Complexin 4 have been proposed to be involved in synaptic light adaptation by limiting synaptic vesicle recruitment and fusion. How this Complexin effect is exerted is unknown. Focusing on rod photoreceptors, we established Complexin 4 as the predominant Complexin in the light-dependent regulation of neurotransmitter release. The number of readily releasable synaptic vesicles is significantly smaller in light than in dark at wildtype compared to Complexin 4 deficient rod photoreceptor ribbon synapses. Electrophysiology indicates that Complexin 4 reduces or clamps Ca^2+^-dependent sustained synaptic vesicle release, thereby enhancing light signaling at the synapse. Complexin 4 deficiency increased synaptic vesicle release and desensitized light signaling. In a quantitative proteomic screen, we identified Transducin as an interactor of the Complexin 4-SNARE complex. Our results provide evidence for a presynaptic interplay of both Complexin 4 and Transducin with the SNARE complex, an interplay that may facilitate the adaptation of synaptic transmission to light at rod photoreceptor ribbon synapses.

## Introduction

Photoreceptors are the specialized sensory neurons of the retina. They absorb photons and transduce this stimulus into electrical and further into chemical signals, which they transmit at their synapses with supreme fidelity to postsynaptic neurons. Photoreceptors face the challenge of detecting light signals with utmost sensitivity over an extremely wide range of signal intensities, e.g., on a bright sunny day as well as during a dark moonless night, with light intensity differences of 10 orders of magnitude. Accordingly, photoreceptors constantly adapt their sensitivity to changes in light intensities. Light adaptation increases their responsiveness to weak input signals, thus improving the signal-to-noise-ratio, and decreases it for strong input signals to remain responsive (Burns and Baylor, 2001; Fain et al., 2001).

Light adaptation is mediated by multiple mechanisms and, in general, the number of adaptive mechanisms employed increases as light gets brighter (Burns and Arshavsky, 2005). Whereas adaptive mechanisms involving signal transduction in the photoreceptor outer segments (OS) have been well described, little is known about the mechanisms that adapt photoreceptor synapse function to changing light intensities. Photoreceptors optimize signal transfer to postsynaptic bipolar and horizontal cells by continuously adjusting their synaptic output to changes in light intensities. As the synaptic output is mediated by the exocytosis of synaptic vesicles (SVs) containing the neurotransmitter glutamate, its continuous adjustment over a physiologically relevant range requires continuous SV release at high rates. This is accomplished by the presence of a specialized electron-dense, plate-like presynaptic structure, the synaptic ribbon, which tethers a large pool of releasable SVs in close vicinity to voltage-gated Ca^2+^ channels at the active zone (Rao-Mirotznik et al., 1995; Heidelberger et al., 2005; Sterling and Matthews, 2005; tom Dieck and Brandstätter, 2006; Moser et al., 2020). The functional development of the photoreceptor ribbon synapse and the major perturbation of photoreceptor synaptic transmission upon genetically induced loss of ribbon biogenesis or active zone anchoring demonstrate the importance of the synaptic ribbon for sustained and spatio-temporally synchronized, high-throughput neurotransmitter release (Dick et al., 2003; Maxeiner et al., 2016; Davison et al., 2022a). As concerns photoreceptor ribbon synapse adaptation, the SV loading density on ribbons in the lizard retina is known to vary depending on the predominant light regime (Jackman et al., 2009). Likewise, photoreceptor synaptic ribbons in mouse retina display light-dependent differences in the number of ribbon-tethered SVs. Here, the ribbon base close to the release site is depleted of SVs in light, when exocytosis is halted, and reloaded with SVs in the dark, when exocytosis is ongoing (Babai et al., 2016).

Complexins are promising candidate proteins involved in the light-dependent replenishment of the ribbon with SVs. They are small, highly charged proteins that control the speed and Ca^2+^ sensitivity of SNARE-mediated SV fusion (McMahon et al., 1995; Reim et al., 2001; López-Murcia et al., 2019). In mouse retina, all four known Complexin (Cplx) isoforms, Cplx1-Cplx4, are present. While Cplx1 and Cplx2 are found at conventional chemical synapses in the retina, photoreceptor ribbon synapses are equipped with Cplx3 and Cplx4 (Reim et al., 2005; Lux et al., 2021). We showed previously for the cone photoreceptor ribbon synapse in mouse retina that (i) Cplx3 and Cplx4 suppress tonic and facilitate evoked transmitter release, (ii) loss of Cplx3 and Cplx4 perturbs ribbon synapse function and differentially impacts ON and OFF pathways of the retina (Reim et al., 2009; Landgraf et al., 2012; Babai et al., 2016), and (iii) Cplx3 and Cplx4 co-regulate the light-dependent replenishment of SVs to the synaptic ribbon of cone photoreceptors (Babai et al., 2016). In the present study, we focused on the rod photoreceptor ribbon synapse. We uncovered a possible interplay of both Cplx4 and the G protein Transducin (G_*t*_) with the SNARE complex that may regulate the light-dependent strength of rod photoreceptor ribbon synapses.

## Materials and methods

### Animals and light regime

Mice were group housed at the animal care facility of the Friedrich-Alexander-Universität Erlangen-Nürnberg (Biologisch-technisches Entwicklungslabor) under a 12 h/12 h light/dark cycle (average illumination of 200 lux; white light; TLD 58W/25 tubes; Philips) with food and water provided *ad libitum*. Killing of the mice to obtain the retinal tissue was approved by the local authorities (Sachgebiet Tierschutzangelegenheiten der Friedrich-Alexander-Universität Erlangen-Nürnberg, AZ TS 7/2023 Tierphysiologie). Adult (2–4 months) male and female C57BL/6J wildtype (WT), Cplx3 knockout (KO), Cplx4 KO and Cplx3/4 double KO (DKO) mice (Reim et al., 2009) were used. For FACS experiments Tg (Rac3-EGFP) JZ58Gsat/Mmcd (Rac3-eGFP) mice, expressing enhanced green fluorescent protein (eGFP) in all cone photoreceptor cells (Gong et al., 2003; Regus-Leidig et al., 2013) were used. These mice were obtained from the Mutant Mouse Regional Resource Center (MMRRC), a National Center for Research Resources (NCRR)-NIH-funded strain repository and were donated to the MMRRC by the NINDS funded GENSAT BAC transgenic project. The Rac3-eGFP construct was generated by inserting an eGFP reporter gene, followed by a polyadenylation sequence, into the bacterial artificial chromosome (BAC) clone RP23-62A17 at the initiating ATG codon of the first coding exon of the Rac3 gene. Consequently, eGFP expression was driven by the regulatory sequence of the Rac3 gene.

All adaptation procedures were carried out without artificial pupil dilation. Light adaptation for quantitative electron microscopy was carried out as described previously (Babai et al., 2016). Briefly, mice were sacrificed during the regular diurnal cycle either 3 h after light onset (9:00 a.m.) or 3 h after light offset (9:00 p.m.). For electrophysiology and the peptide-based affinity purification approach, both light adaptation (∼ 3 h) and subsequent tissue preparation were performed under white light (∼ 500 lux). For proximity ligation assays, mice were sacrificed after 3 h dark adaptation or after bright light adaptation (∼ 2000 lux for 1 h), thus exceeding the necessary 250 lux and time for successful Transducin translocation (Sokolov et al., 2002; Kassai et al., 2005). All dark-adapted samples were obtained under dim red light.

### Immunocytochemistry

Preparation of mouse retinae for cryostat sections and antibody incubation for light microscopic immunocytochemistry and proximity ligation assays (PLA) were done as described previously with minor modifications (Regus-Leidig et al., 2013). Briefly, mice were deeply anesthetized by inhalation of isoflurane (Abbott Laboratories, Chicago, IL, USA) and sacrificed by cervical dislocation. For cryostat sections, the eyes were opened and fixed in the eyecup in 4% paraformaldehyde (PFA) in 1 × PBS (pH 7.4) for 30 min at room temperature (RT). After washing, the eyes were cryoprotected in 10%, 20% and 30% (w/v) sucrose in PBS and mounted in Tissue-Tek O.C.T. freezing medium (Sakura Finetek Germany, Staufen, Germany). Vertical cryostat sections (14 μm thick) were cut with a cryostat (CM3050 S, Leica Microsystems, Wetzlar, Germany) and collected on glass slides. The sections were washed in PBS and blocked in blocking solution [10% normal goat serum (NGS), 1% bovine serum albumin (BSA), 0.5% Triton X-100 in PBS] for 60 min at RT. Primary antibodies were diluted in antibody solution (3% NGS, 1% BSA, 0.5% Triton X-100 in PBS) and incubation was performed overnight at 4°C. Then, the samples were washed three times with PBS and incubated with secondary antibodies and DAPI (0.1 μg/ml) diluted in antibody solution for 2 h at RT.

For vibratome sections, the eyes were opened and fixed in the eyecup in 4% PFA and 0.02% picric acid in 1 × phosphate buffer (pH 7.4) for 30 min. After washing, cryoprotection and retina extraction, the retinae were cracked three times using a liquid nitrogen cooled copper block. Retinae were embedded in 3% low melting agarose and cut with a vibratome (VT1000S, Leica Microsystems) to obtain 60 μm thick vertical sections. The free-floating sections were subjected to the antibody staining protocol described above with blocking for 90 min at RT, incubation with primary antibodies for 3 days at 4°C and incubation with secondary antibodies for 3 h at RT. After final washing steps, the samples were mounted on glass slides using Aqua-Poly/Mount (Polysciences).

### Proximity ligation assay

Tissue preparation and cryostat sectioning of mouse retinae was done as described above. Samples for PLA experiments of dark- and light-adapted C57BL/6J, Cplx4 WT and Cplx4 KO and Cplx3/4 DKO mice were collected on the same slides to ensure equal treatment throughout the experiment. Washing, blocking and primary incubation was performed as described above. The next day, PLA experiments were performed according to the manufacturer’s instructions (NaveniFlex MR, Navinci Diagnostics, Uppsala, Sweden) including washing steps between all PLA specific reactions using TBS-T (0.05% Tween 20 in 1 × TBS). Briefly, after incubation with PLA probes, the reactions A (activation of oligonucleotides), B (ligation of probes in close proximity) and C (rolling circle amplification and fluorescent probe binding) were carried out. Subsequently, sections were rinsed in 1 × TBS and incubated with secondary antibodies and DAPI (0.1 μg/ml) diluted in antibody solution for 1 h at RT. After final washing steps in 1 × TBS the samples were rinsed in 0.1 × TBS and mounted using Aqua-Poly/Mount (Polysciences).

### Antibodies

The following primary antibodies were used for immunocytochemistry: mouse anti-Alpha Transducin (1:50; catalog #F-12, sc-518142), rabbit anti-Alpha Transducin (1:2000; catalog #K-20, sc-389, both Santa Cruz Biotechnology, Dallas, TX, USA), rabbit G Protein Subunit Gamma Transducin 1 (1:10,000; catalog #PA5-49350, Thermo Fisher Scientific, Waltham, MA, USA), mouse anti-Complexin 3 (1:500; catalog #122 311), rabbit anti-Complexin 4 (1:40,000; catalog #122 402), mouse anti-Complexin 4 (1:50,000; catalog #122 411), mouse anti-VAMP2 (1:20,000; catalog #104 211, all Synaptic Systems GmbH, Göttingen, Germany). Fluorophore-coupled secondary antibodies were used for visualization of primary antibodies: Alexa^®^Fluor Plus 488/647-conjugated goat anti-mouse and goat anti-rabbit IgG (1:500/1:200; catalog #A32731, A32733, A32723, A32728) and Alexa^®^Fluor 568-conjugated goat anti-rabbit and mouse IgG (1:500; catalog #A11036, A11004, all Thermo Fisher Scientific). Cell nuclei were labeled with DAPI (0.1 μg/ml).

The following primary antibodies were used for Western Blot (WB) experiments: mouse anti-Syntaxin 1AB (1:10,000; catalog #110 001), mouse anti-SNAP25 (1:5000; catalog #111 001), mouse anti-Synaptobrevin 2 (1:7500; catalog #104 211), rabbit anti-Syntaxin 3 (1:1000; catalog #110 033), mouse Munc18-1 (1:1000; catalog #116 011, all Synaptic Systems GmbH), rabbit anti-Alpha Transducin (1:1000; catalog #PAS-26784), rabbit G Protein subunit Beta Transducin 1 (1:5000; catalog #PAS-30046, both Thermo Fisher Scientific). To detect specific signals on the membranes by using the Odyssey Infrared Imaging System (LI-COR Biosciences, Bad Homburg, Germany) the following fluorophore-coupled secondary antibodies were used: goat anti-mouse and goat anti-rabbit IgG Alexa^®^Fluor680 RD (1:5000; catalog #A-21058 and A-21109, Thermo Fisher Scientific) as well as goat anti-mouse and goat anti-rabbit IgG IRDye800 CW (1:5000; catalog #926-32210 and 926-32211, LI-COR).

### Light microscopy and analysis of immunofluorescence data

For light microscopical analysis, labeled sections were examined with an Axio Imager.M2 equipped with an ApoTome.2 module or a Laser Scanning Microscope 710 with corresponding imaging modules (Carl Zeiss AG, Oberkochen, Germany). Images were acquired using a 20 × (0.8 NA, Apochromat) or a 63 × (1.4 NA oil immersion, Plan Apochromat) objective (both Carl Zeiss AG) as stacks of multiple optical sections and projections were calculated with ZEN blue or ZEN black software (Carl Zeiss AG). Images were adjusted for contrast and brightness using Photoshop CS6 (Adobe Systems, San Jose, CA, USA) and arranged using CorelDRAW 2021 (Corel Corporation, Ottawa, ON, Canada). Every comparative PLA experiment was carried out on one slide, ensuring equal treatment of the samples. Three experiments with samples from three mice of each condition were analyzed. The corresponding images were taken with the same settings and adjusted equally in brightness and contrast. The different regions of interest were determined manually, and fluorescence intensities were measured using ImageJ (NIH, Bethesda, MA, USA). The obtained information was summarized in Excel (Microsoft Corporation, Redmond, WA, USA), PLA signal intensity was normalized to the mean of each slide. Graphs were created using GraphPad Prism 9.0 (GraphPad Software Inc., San Diego, CA, USA) and arranged together with images using CorelDRAW 2021 (Corel Corporation).

### Fluorescence-activated cell sorting of photoreceptor cells

Photoreceptors were sorted as described previously (Davison et al., 2022b). Briefly, retinae of Rac3-eGFP mice were dissociated by papain digestion and subsequent trituration. Cone photoreceptors were sorted by cone photoreceptor-specific fluorescence of Rac3-eGFP mice. For the sorting of unlabeled rod photoreceptors, we used forward/sideward scatter caused by the high backscatter of heterochromatin in the core of the rod photoreceptor nucleus in an approach adapted from Feodorova et al. (2015). Cells were sorted in a FACS Aria III (BD Biosciences, San Jose, CA, USA) with an 85 μm nozzle. For RNA analysis, cells were directly collected in RLT buffer (Qiagen, Hilden, Germany) containing 1% β-Mercaptoethanol. For protein analysis by gel electrophoresis, cells were directly collected in 1 × PBS supplemented with 5 mM EDTA. For the figure, images were created using FlowJo Software Version 10.8.1 for Windows (Becton, Dickinson and Company, Ashland, OR, USA) and arranged with CorelDRAW 2021 (Corel Corporation).

### RT-qPCR

Total RNA of sorted photoreceptors was isolated with the RNeasy Micro Kit (Qiagen, Hilden, Germany). RNA was reverse transcribed to cDNA using the iScript cDNA Synthesis Kit (Bio-Rad Laboratories, Munich, Germany). RT-qPCR was performed with 59°C annealing temperature in a volume of 12.5 μl using 1 μl of the prepared cDNA and 0.3 μl of each primer (of the 10 pM working solution) per reaction. Gene expression was normalized to beta actin (Actb), glyceraldehyde 3-phosphate dehydrogenase (Gapdh) and importin 8 (Ipo8) and quantified using CFX Manager 3.1 Software (Bio-Rad Laboratories) and Excel (Microsoft Corporation). The graphs were created using GraphPad Prism 9.0. The following specific primer pairs were used: Rhodopsin (Rho) forward primer (F): 5′-GTCATCTACATCATGTTGAAC-3′; Rho reverse primer (R): 5′-ATCTCCCAGTGGATTCTT-3′; short-wave-sensitive opsin 1 (Opn1sw) F: 5′-CTCTTCTGCATCTTCTCT-3′; Opn1sw R: 5′-AGGGTTTACAGATGACAA-3′; Cplx3 F: 5′-GAAGAG TACGAGGAGTATC-3′; Cplx3 R: 5′-CTTCCTCTGTGTGAAC TG-3′; Cplx4 F: 5′-GGCTAAAGGGATGACTAG-3′; Cplx4 R: 5′-CTCTCTCCATCTTCTCTTC-3′; Actb F: 5′-TTCCTCCCTG GAGAAGAG-3′; Actb R: 5′-CACTGTGTTGGCATAGAG-3′; Gapdh F: 5′-CAACTTTGTCAAGCTCATT-3′; Gapdh R: 5′-TCTGGGATGGAAATTGTG-3′; Ipo8 F: 5′-TGTCACC ATGTTCTTCAGGTAGAT-3′; Ipo8 R: 5′-TATTAATTTTGCCCC CAGCTT-3′.

### Gel-based proteome analysis of FACS-sorted photoreceptor cells

To the sorted cell samples in PBS/5 mM EDTA (∼50,000 cells in ∼500 μl), SDS was added at a final concentration of 0.5% and cell lysis was performed for 30 min at 22°C under gentle shaking, followed by a 2 min treatment in an ultrasonic bath. Samples were precipitated by methanol/chloroform and the pellets were pooled in 45 μl SDS sample buffer to generate a cone and a rod photoreceptor cell sample, each corresponding to 187,000 cells. Proteins were separated on a pre-cast Tris-glycine 4–12% gradient gel (TG PRiME, Serva Electrophoresis GmbH, Heidelberg, Germany), stained with colloidal Coomassie, and entire gel lanes were excised as 24 equally sized gel bands. Automated tryptic in-gel digestion of proteins, separation of tryptic peptides by liquid chromatography (LC), and label-free quantification of proteins by mass spectrometry (MS) were performed as described (Sondermann et al., 2019).

### Tissue preparation and quantitative electron microscopy

Tissue preparation and quantitative electron microscopy was performed as described previously (Babai et al., 2016). For optimal tissue preservation, retinae were fixed in 4% PFA and 2.5% glutaraldehyde for 2 h at RT. Tissue contrasting was performed by incubation in 1.5% potassium ferrocyanide and 2% osmium tetroxide in cacodylate buffer for 1.5 h. Retinal tissue was dehydrated with an ethanol series and propylene oxide with 0.5% uranyl acetate added at the 70% ethanol step, before embedding in Renlam resin (Serva). Ultrathin sections (60 nm) were stained with uranyl acetate and lead citrate and examined using a Zeiss EM10 electron microscope. Images were taken with a Gatan SC1000 Orius TM CCD camera in combination with the Digital Micrograph TM software (Gatan, Pleasanton, CA, USA). Images were adjusted for contrast and brightness using Photoshop CS6 (Adobe Systems). For each experimental condition, we prepared random ultrathin sections of retinae from three mice and examined between 100 and 300 rod photoreceptor terminals. For the analysis of the number of ribbon-tethered SVs in single ultrathin sections, we chose only photoreceptor ribbon synaptic complexes with a rod-shaped presynaptic ribbon and with both the arciform density and the dyadic or triadic arrangement of the invaginating postsynaptic elements visible in the section plane. The first row of SVs (∼30 nm distance to the ribbon) along the entire height of the ribbon and within the basal 100 nm of the ribbon was counted in single sections. The height of the synaptic ribbons was determined by measuring the perpendicular extension of the ribbon into the cytosol. The quantification results were gathered using Excel (Microsoft Corporation) and visualized with GraphPad Prism 9.0. Schematic representations were created with BioRender.com and panels were arranged using CorelDRAW 2021 (Corel Corporation).

### Peptide-based affinity purification approach

Cplx-derived peptides (see Figure 4A for amino acid sequences) were synthesized by standard solid-phase peptide synthesis using fluorenylmethoxycarbonyl (Fmoc) chemistry. In Cplx4-derived peptides, the internal cysteine residue was replaced by α-aminobutyric acid, a commonly used non-reactive cysteine analog with similar polarity (Ferrer et al., 1992), to ensure directed immobilization of the peptides via their N-terminal cysteine residues. Peptides were coupled to sulfhydryl-reactive beads (SulfoLink^®^ Coupling Resin, Thermo Fisher Scientific) according to the manufacturer’s instructions. Briefly, 100 μl beads were washed four times with coupling buffer (5 mM EDTA-Na, 50 mM Tris pH 8.5) and incubated with 250 μl peptide solution (1.5 μg/μl in coupling buffer) for 1 h at room temperature while rotating. After four washes with coupling buffer, the beads were incubated as above with 50 mM cysteine in coupling buffer to block remaining reactive sites. Beads to be used as negative control were only saturated with cysteine. Beads were washed five times with 1 M NaCl, three times with PBS, and stored for short-term in PBS/0.05% NaN_3_ at 4°C until further use. For affinity purification experiments, retinae were isolated and homogenized in 0.32 M sucrose solution with protease inhibitors (17 μg/ml PMSF, 1 μg/ml aprotinin and 0.5 μg/ml leupeptin) using a glass teflon homogenizer. Retinae from 10 mice and 100 μl sucrose solution were used per affinity matrix (i.e., per immobilized peptide) in the experiment. The retina homogenate was adjusted to a protein concentration of 2 mg/ml with solubilization buffer (1% NP40, 150 mM NaCl, 1 mM EGTA, 2 mM MgCl_2_, 1 mM DTT, 10 mM HEPES pH 7.4, protease inhibitors) and incubated on a rotating wheel for 15 min at 4°C. Insoluble material was removed by ultracentrifugation at 356,200 × g for 15 min at 4°C. The clear supernatant (referred to as “load”) was added to 50 μl peptide-coupled agarose beads and incubated for 3 h at 4°C while rotating. Beads were washed five times with solubilization buffer and residual supernatants removed carefully. For subsequent immunoblotting, beads were resuspended in 100 μl 1 × SDS sample buffer and boiled at 95°C for 5 min. Gel electrophoresis with self-cast polyacrylamide gels and protein transfer via tank blotting was performed using standard protocols. For subsequent in-solution digestion according to the filter-aided sample preparation (FASP) procedure, beads were resuspended in 250 μl FASP solubilization buffer (7 M urea; 2 M thiourea; 2% CHAPS; 10 mM DTT, 0.1 M Tris pH 8.5), incubated for 20 min at RT, and centrifuged at 16,000 × *g*. The supernatant was subjected to FASP, followed by quantitative MS analysis. Graphs were created using GraphPad Prism 9.0 and arranged together with the images using Adobe Illustrator 2020.

### Gel-free proteome analysis of affinity-purified samples

Eluates from affinity purification experiments in FASP solubilization buffer were directly subjected to in-solution digestion and label-free protein quantification as described previously (Ambrozkiewicz et al., 2018). Briefly, protein fractions were digested with trypsin according to a CHAPS-based FASP protocol in centrifugal filter units (30 kDa MWCO, Merck Millipore). Tryptic peptides were recovered by centrifugation and extracted with 40 μl of 50 mM ammonium bicarbonate and 40 μl of 1% trifluoroacetic acid (TFA). Combined flow-throughs were spiked with 10 fmol/μl yeast enolase-1 tryptic digest standard (Waters Corporation) for quantification purposes and directly subjected to LC-MS-analysis. Nanoscale reversed-phase UPLC separation of tryptic peptides was performed over 120 min at a flow rate of 300 nl/min with a gradient comprising two linear steps of 3–35% mobile phase B (acetonitrile containing 0.1% formic acid) in 105 min and 35–60% mobile phase B in 15 min. MS analysis on a quadrupole time-of-flight mass spectrometer (Synapt G2-S, Waters Corporation) was performed in the ion mobility-enhanced data-independent acquisition mode with drift time-specific collision energies. Using Waters ProteinLynx Global Server with previously described settings (Ambrozkiewicz et al., 2018), data were searched against a custom database compiled by adding the sequence information for yeast enolase 1 (UniProtKB/Swiss-Prot accession number P00924), porcine trypsin (P00761), Isoform 3B of Syntaxin-3 (Q64704-2), and Isoform 2 of C-terminal-binding protein 2 (RIBEYE, P56546-2) to the UniProtKB/Swiss-Prot mouse proteome (release 2019_11, 17027 entries) and by appending the reversed sequence of each entry to enable the determination of false discovery rate (FDR). ISOQuant^1^ was used for post-identification analysis including TOP3 quantification of proteins (Ambrozkiewicz et al., 2018). FDR for both peptides and proteins was set to 1% threshold and only proteins represented by at least two peptides (one of which unique) were quantified in fmol. Protein abundance in eluates from Cplx4 WT and Cplx4 M affinity matrices was assessed in technical replicates at digestion level from three independent experiments. For the classification of interaction partners according to gene ontology (GO)-terms, the PANTHER classification system (Thomas et al., 2022) was used.^2^

### Slice preparation and electrophysiology

The retinal slicing procedure was introduced previously (Feigenspan and Babai, 2017). Briefly, Cplx4 WT and Cplx4 KO mice were anaesthetized with isoflurane (3%) and euthanized by cervical dislocation. The retina was removed from the eye, placed in Ames’ medium (Sigma-Aldrich, Munich, Germany) and cut into quarters. Then, the retina was mounted flat in 1.8% low melting agarose dissolved in Ames’ medium and a horizontal cut was made at the level of the outer plexiform (OPL) layer, using a vibratome (Leica Microsystems, Wetzlar, Germany). The prepared retinal slices were visualized using a 63 × water immersion objective (Zeiss, Jena, Germany) on a fixed stage microscope (Zeiss Axio Examiner). Whole-cell patch-clamp recordings were made targeting rod photoreceptor somas, which are located close to the OPL (Grabner et al., 2015). Recordings were performed using an EPC-10 patch-clamp amplifier (Heka Elektronik, Lambrecht, Germany), low-pass filtered at 2.9 kHz using a built-in Bessel filter and digitized at 10 kHz with the Patchmaster software (HEKA Elektronik GmbH, Reutlingen, Germany). All recordings were made at RT (22–24°C) under room light conditions. To maintain the light-adapted condition, retinal slices were constantly exposed to the transmitted light path of the microscope. During recordings, slices were continuously perfused (∼1 ml/min) with bubbled (95% O_2_/5% CO_2_) extracellular solution containing (in mM): 116 NaCl, 22.6 NaHCO_3_, 1.25 NaH_2_PO_4_, 2.5 KCl, 2 CaCl_2_, 1 MgCl_2_, 10 glucose, 5 HEPES, 1 ascorbic acid, and 2 sodium pyruvate, adjusted to pH 7.4. Patch pipettes were pulled from borosilicate glass (Sutter Instruments, Novato, CA) to a final resistance of 9 to 12 MΩ. I_AGlu_ recordings were made using a cesium/thiocyanate-based intracellular solution which contained (in mM): 82.5 potassium thiocyanate, 30 Cs-gluconate, 13.3 Cs-Glutamate, 5 EGTA, 11 TEA-Cl, 11.6 HEPES, 3 Mg^2+^-ATP, 1.8 Mg^2+^-GTP, 0.67 CaCl_2_, and 0.67 MgCl_2_ (pH 7.2). Recordings with an access resistance exceeding 55 MΩ were excluded. Membrane potential was corrected for a liquid junction potential of + 7 mV. The liquid junction potential was calculated according to the stationary Nernst-Planck equation using LJPcalc software^3^ (Marino et al., 2014). I_AGlu_ events recorded from rod photoreceptors were analyzed using Mini Analysis software (Synaptosoft, Fort Lee, NJ, USA). For clearness of illustration, current traces were low-pass filtered at 1 kHz.

### Experimental design and statistical analysis

Data are presented as mean ± SD (SE for Figure 3) with sample sizes indicated as n in the figure legends and exact *p*-values given in the text. For comparison of two means, we performed two-tailed unpaired *t*-tests if data were normally distributed. The Mann-Whitney *U*-test was used for comparisons of data that could not be described by a normal distribution. *F*-tests were performed to detect differences in variances. Welch correction of the *t*-test was applied if two groups displayed significantly different variances. For multiple comparison, we performed a one-way-ANOVA with Tukey’s multiple comparison *post-hoc* test. The Brown-Forsythe test showed no differences in variances, hence no correction was needed. The comparison between event amplitude distributions was performed using a Two-Sample Kolmogorov-Smirnov Test. Data were tested with the Shapiro-Wilk test for Gaussian distribution. In all cases, statistical significance was accepted at the *p* < 0.05.

## Results

### Cplx4 is the predominant Cplx isoform in mouse rod photoreceptors

In previous work, based on immunocytochemistry, we described the presence of both Cplx3 and Cplx4 in mouse cone photoreceptors and of Cplx4 only in mouse rod photoreceptors (Reim et al., 2005; Landgraf et al., 2012). Recently, the presence of both Cplx3 and Cplx4 mRNAs in mouse rod photoreceptors has been reported (Bhoi et al., 2021). Since the aim of the present study is to investigate the role of Cplx4 in mouse rod photoreceptors, we investigated the presence of Cplx3 and Cplx4 at the mRNA level and at the protein level using a proteomic approach in addition to immunocytochemistry.

**FIGURE 1:**
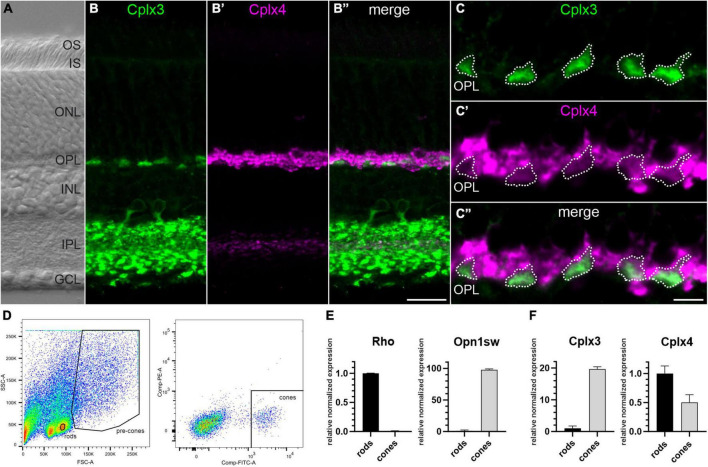
Cplx4 is the predominant Cplx isoform in mouse rod photoreceptors. **(A)** Nomarski micrograph of a vertical vibratome section through mouse retina showing the different retinal layers. **(B–C”)** Fluorescence micrographs of vertical vibratome sections through mouse retinae double stained with anti-Cplx3 and Cplx4 antibodies. A higher-power view of the Cplx3 and Cplx4 staining in the outer plexiform layer (OPL) is shown in **(C–C”)**. **(D)** Fluorescence activated cell sorting (FACS) strategy for sorting of rod and cone photoreceptors. RT-qPCR analysis of Rhodopsin (Rho) and short wave sensitive opsin 1 (Opn1sw) **(E)**, and Cplx3 and Cplx4 **(F)** in sorted rod and cone photoreceptors. Bar graphs display mean relative expression levels normalized to rod photoreceptors (mean ± SD in 6 animals). OS, outer segments; IS, inner segments; ONL, outer nuclear layer; INL, inner nuclear layer; IPL, inner plexiform layer; GCL, ganglion cell layer. Scale bar = 20 μm in **(B”)** for **(A–B”)** and 5 μm in **(C”)** for **(C–C”)**.

Immunocytochemical double labeling of vertical retinal vibratome sections with anti-Cplx3 and anti-Cplx4 antibodies showed immunoreactivity for the two Cplxs in both synaptic layers of the retina, the inner plexiform layer (IPL) and the outer plexiform layer (OPL), but with different distributions (Figures 1A–B″). Cplx3 immunoreactivity in the IPL represents amacrine cell processes and rod bipolar cell terminals, and in the OPL cone photoreceptor terminals (Figures 1B, C, C″). Cplx4 immunoreactivity in the IPL represents cone bipolar cell terminals and in the OPL strongly and weakly labeled rod and cone photoreceptor terminals, respectively (Figures 1B′, C′, C″; see also Reim et al., 2005; Landgraf et al., 2012). In summary, these results confirm that cone photoreceptor ribbon synapses contain Cplx3 and Cplx4, while Cplx4 is the predominant isoform in rod photoreceptor ribbon synapses.

Next, we performed RT-qPCR analyses of sorted rod and cone photoreceptors (see Materials and methods). To verify our sorting strategy (Figure 1D), we tested the sorted photoreceptor samples for the rod photoreceptor-specific pigment rhodopsin (Rho) and the cone photoreceptor-specific pigment short-wave-sensitive opsin 1 (Opn1sw). We found high Rho vs. Opn1sw mRNA levels in rod vs. cone photoreceptor samples, with hardly any cross contamination, which validates our sorting strategy (Figure 1E). Expression of Cplx3 and Cplx4 mRNAs in sorted photoreceptors proved that cone photoreceptors express both isoforms, whereas Cplx4 mRNA is the predominant Cplx transcript in rod photoreceptors (Figure 1F).

Finally, we separated proteins of sorted photoreceptor samples by gel electrophoresis (Supplementary Figure 1A). Instead of immunoblotting, we quantified proteins by mass spectrometry (MS) as an untargeted approach to assess the expression of Cplx3 and Cplx4 together with selected marker proteins (Supplementary Figure 1B and Supplementary Data 1). We confirmed Opn1sw as a cone photoreceptor-specific marker that was not detectable in rod photoreceptors. In addition, we detected the medium-wave-sensitive opsin 1 (Opn1mw) and cone-type Transducin alpha (Gnat2) and gamma subunits (Gngt2) in our cone photoreceptor sample. For Rho as a marker for rod photoreceptors, we had to choose an alternative because the Rho-containing rod photoreceptor outer segments were lost during the sorting process. We detected the rod photoreceptor-specific marker proteins Phosphodiesterase 6A (Pde6a) and rod-type Transducin alpha (Gnat1) and gamma subunits (Gngt1) in our rod photoreceptor sample (Supplementary Figure 1B and Supplementary Data 1). While Cplx3 was not detectable in rod photoreceptors, confirming its cone photoreceptor specificity, Cplx4 showed a rod-to-cone photoreceptor ratio of ∼2.2 (Supplementary Data 1), consistent with the mRNA data (Figure 1F).

In summary, our immunolabeling and cell sorting experiments show that Cplx4 is the predominant Cplx isoform in rod photoreceptors.

### Cplx4 is involved in the light-dependent replenishment of SVs to the rod photoreceptor synaptic ribbon

**FIGURE 2:**
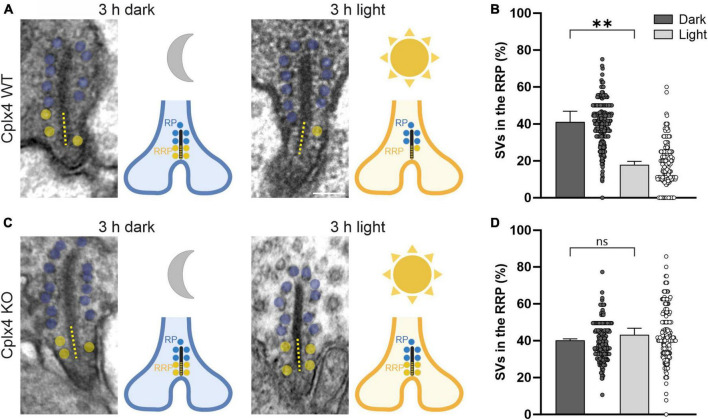
Cplx4 is involved in the light-dependent replenishment of SVs to the rod photoreceptor synaptic ribbon. Electron micrographs and schematic representations of rod photoreceptor ribbons from Cplx4 WT **(A)** and Cplx4 KO **(C)** retinae after 3 h of dark and 3 h of light adaptation. Ribbon-tethered SVs within the basal 100 nm of the ribbon (dotted line), representing the readily releasable SV pool (RRP), are highlighted in yellow, the SVs tethered higher up on the ribbon, representing the reserve SV pool (RP), in blue. Percentage of SVs in the RRP of rod photoreceptor ribbons from Cplx4 WT **(B)** and Cplx4 KO **(D)** retinae after 3 h of dark (dark gray) and 3 h of light (light gray) adaptation. Bar graphs display mean ± SD in 3 animals; p**< 0.005, ns, not significant, *t*-test. Scatter plots display single analyzed ribbons (number of analyzed ribbons for dark WT = 302, light WT = 262, dark KO = 257, light KO = 176). Schematic representations in **(A,C)** were created with BioRender.com. Scale bar = 100 nm in **(A)** for **(A,C)**.

The number of SVs tethered to the base of photoreceptor synaptic ribbons, close to the release site, determines the size of the readily releasable SV pool (RRP) (Thoreson, 2021). This varies with the adaptation state in wildtype (WT) but not in Cplx3/4 double knockout (KO) photoreceptors (Babai et al., 2016). Focusing on rod photoreceptors and the involvement of Cplx4 in the regulation of the size of the RRP, we used quantitative electron microscopy and examined the number of SVs tethered to rod photoreceptor synaptic ribbons in dark- and light-adapted Cplx4 WT mice and their corresponding Cplx4 KO littermates. For the analysis of ribbon-tethered SVs we chose only cross-sectioned rod photoreceptor ribbon synaptic sites with a presynaptic ribbon and the dyadic or triadic arrangement of postsynaptic elements (Figures 2A, C). Cplx4 WT and KO mice were dark- or light-adapted for 3 h and the number of tethered SVs (within ∼30 nm of the ribbon) along the entire height of the synaptic ribbon and within 100 nm of the ribbon base adjacent to the active zone was quantified in single ultrathin sections. We chose 100 nm as a standard to define anatomically the RRP, which represents the bottom two rows of ribbon-tethered SVs (Thoreson, 2021). The remaining distal part of the ribbon ≥100 nm away from the ribbon base was hence defined as the reserve SV pool (RP) (Thoreson, 2021). The number of SVs in the RRP is given as the percentage of the total SV number on the synaptic ribbon (100%) in a given section.

Cplx4 WT rod photoreceptor synaptic ribbons showed a significant reduction in the number of SVs in the RRP after light adaptation (dark ∼41.0%, light ∼17.9%; *p* = 0.003, *t*-test) (Figures 2A, B). In contrast, Cplx4 KO rod photoreceptor synaptic ribbons showed no significant adaptation-dependent changes in RRP SV number (dark ∼40.3%, light ∼43.2%; *p* = 0.23, *t*-test) (Figures 2C, D). Importantly, the height of the rod photoreceptor synaptic ribbons and the SV number of the RP did not differ between genotypes and adaptation conditions (Table 1).

**TABLE 1:** Ribbon synapse parameters presented as mean values ± SD in 3 animals; ANOVA.

	Cplx4 WT		Cplx4 KO		
	**Dark**	**Light**	**Dark**	**Light**	**ANOVA (p)**
Ribbon height (nm)	204 ± 18	225 ± 17	215 ± 17	206 ± 40	0.75
Total SVs	10.8 ± 1.3	9.4 ± 0.5	11.3 ± 0.5	10.8 ± 2.0	0.32
RP	6.4 ± 0.7	7.7 ± 0.5	6.8 ± 0.3	6.3 ± 1.3	0.19
RRP	4.4 ± 0.9	1.6 ± 0.2	4.4 ± 0.2	4.5 ± 0.8	0.0013

SV, synaptic vesicle; RP, reserve SV pool; RRP, readily releasable SV pool.

In conclusion, the data from our ultrastructural study show that Cplx4 is involved in the light-dependent regulation of the RRP size at rod photoreceptor ribbon synapses.

### Cplx4 reduces SV release in light-adapted rod photoreceptor ribbon synapses in a Ca^2+^-dependent manner

**FIGURE 3:**
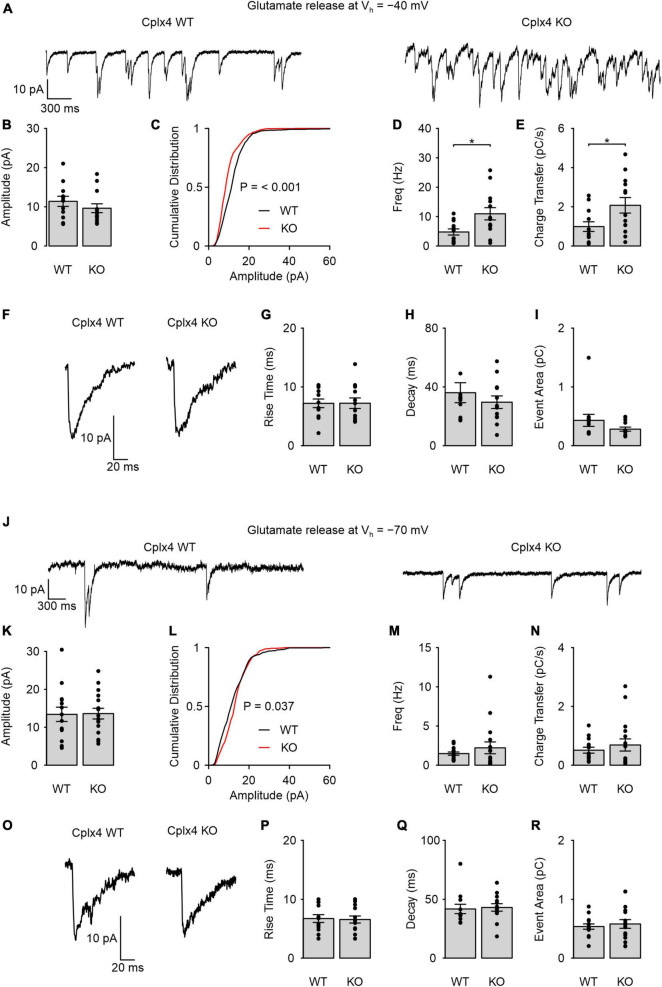
Cplx4 reduces SV fusion in light-adapted rod photoreceptor ribbon synapses in a Ca^2+^-dependent manner. **(A–R)** Patch-clamp recordings of sustained activity in Cplx4 WT and KO mouse rod photoreceptors. **(A)** Representative traces of I_AGlu_ events recorded from rod photoreceptors at *V*_h_ = –40 mV in Cplx4 WT and Cplx4 KO mice. **(B)** Amplitude of I_AGlu_ events; Mann-Whitney *U*-test. **(C)** Cumulative probability plot of I_AGlu_ event amplitude (bin = 1 pA, 521 – 918 events, two-sample Kolmogorov-Smirnov test). **(D)** Frequency of I_AGlu_ events; unpaired *t*-test. **(E)** Charge transfer rate of I_AGlu_ events; unpaired *t*-test. **(F)** Representative I_AGlu_ events in Cplx4 WT and Cplx4 KO mice at *V*_h_ = –40 mV. **(G)** 10:90% rise time of I_AGlu_ events; unpaired *t*-test. **(H)** Tau of a single exponential fit to the I_AGlu_ event decay; Mann-Whitney *U*-test. **(I)** I_AGlu_ event area; Mann-Whitney *U*-test. **(J)** Representative traces of I_AGlu_ events recorded from rod photoreceptors at V_h_ = –70 mV in Cplx4 WT and Cplx4 KO mice. **(K)** Amplitude of I_AGlu_ events; unpaired *t*-test. **(L)** Cumulative probability plot of I_AGlu_ event amplitude (bin = 1 pA, 421–548 events, two-sample Kolmogorov-Smirnov test). **(M)** Frequency of I_AGlu_ events; Mann-Whitney *U*-test. **(N)** Charge transfer rate of I_AGlu_ events; Mann-Whitney *U*-test. **(O)** Representative I_AGlu_ events in Cplx4 WT and Cplx4 KO mice at *V*_h_ = –70 mV. **(P)** 10:90% rise time of I_AGlu_ events; unpaired *t*-test. **(Q)** Tau of a single exponential fit to the I_AGlu_ event decay; Mann-Whitney *U*-test. **(R)** I_AGlu_ event area; unpaired *t*-test. All bar graphs display mean ± SE in 3–5 animals. Data points in bar graphs represent individual cells (*n* = 12–16 cells). p* < 0.05.

To assess the consequences of Cplx4 loss on rod photoreceptor ribbon synapse function, we performed whole-cell patch-clamp recordings of light-adapted Cplx4 WT and KO rod photoreceptors. To detect SV release, we measured glutamate transporter-associated anion currents (I_AGlu_), an approach that has been used in several studies to evaluate SV release in conventional and ribbon type synapses (Hays et al., 2020; Davison et al., 2022a). Rod photoreceptor membrane potential (V_h_) was held at −40 mV, where voltage-gated Ca^2+^ channels at the active zone are active and trigger sustained SV release. Rod photoreceptors exhibited sustained SV release in both Cplx4 WT and KO mice (Figure 3A). Individual release events of Cplx4 WT and KO rod photoreceptor ribbon synapses showed an average amplitude of ∼10 pA (Figure 3B). However, the cumulative amplitude distribution did show a left shift relative to the WT condition (Figure 3C). Interestingly, Cplx4 KO rod photoreceptors showed an increase in both synaptic event frequency and in charge transferred by I_AGlu_ events over time (Figures 3D, E). This indicates that Cplx4 KO caused an increase in Ca^2+^-dependent SV release in light-adapted rod photoreceptors. To further investigate SV release in Cplx4 WT and KO rod photoreceptor ribbon synapses, we compared the rise time, decay time, and area of individual I_AGlu_ events but found no effect of Cplx4 KO on these synaptic parameters (Figures 3F–I).

We further measured SV release from rod photoreceptors at a hyperpolarized membrane potential (*V*_h_ = −70 mV), when voltage-sensitive Ca^2+^ channels are not activated. In this condition, we observed significantly fewer I_AGlu_ events in both genotypes (Figure 3J) as compared to SV release at *V*_h_ = −40 mV (WT I_AGlu_ event frequencies at −40 vs. −70 mV: *p* = 0.009, *t*-test; KO I_AGlu_ event frequencies at −40 vs. −70 mV: *p* < 0.0001, Mann-Whitney-*U*-test). I_AGlu_ event amplitude was not significantly different between Cplx4 WT and KO rod photoreceptors (Figure 3K). Furthermore, we only found a marginally significant change in the cumulative amplitude distribution at events below ∼17 pA for Cplx4 KO photoreceptors (Figure 3L). Both event frequency and charge transferred by I_AGlu_ over time were not significantly different between Cplx4 WT and KO rod photoreceptors (Figures 3M, N). These results indicate that Cplx4 KO does not affect SV release when voltage-gated Ca^2+^ channels are not activated. As with *V*_h_ = −40 mV, we found no effects of Cplx4 KO on the rise time, decay time, and area of individual I_AGlu_ events at for *V*_h_ = −70 mV (Figures 3O–R).

In summary, our patch-clamp experiments indicate that Cplx4 acts in light-adapted rod photoreceptors to reduce or clamp Ca^2+^-dependent sustained synaptic vesicle release, but does not affect spontaneous release when Ca^2+^ channels are not activated.

### A peptide-based affinity purification approach to study Cplx4-SNARE complex interactors in the retina

**FIGURE 4:**
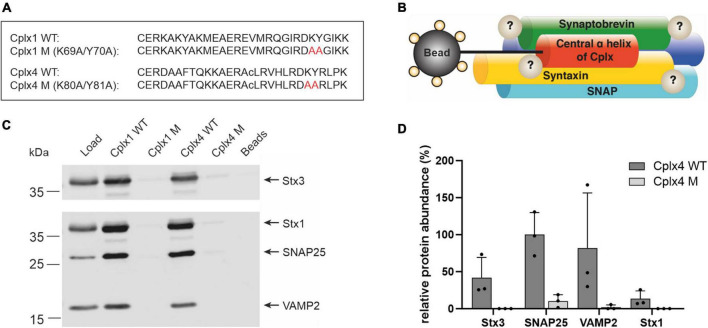
A peptide-based affinity purification approach to study Cplx4-SNARE complex interactors in the retina. **(A)** Sequence of the synthetic Cplx1 and Cplx4 WT peptides and their respective SNARE binding-deficient mutant versions. The amino acid exchanges K69A/Y70A in Cplx1 M and K79A/Y80A in Cplx4 M are labeled in red (amino acid numbering refers to the full-length proteins). Lower case c as single-letter code for the internal cysteine residue in Cplx4-based peptides indicates replacement by α-aminobutyric acid, a commonly used non-reactive cysteine analog with similar polarity. **(B)** Schematic illustration of the Cplx peptide-based affinity purification approach. **(C)** Immunodetection of the SNARE proteins Syntaxin 1 (Stx1), SNAP25, VAMP2 and Syntaxin 3 (Stx3) after affinity purification with Cplx wildtype (Cplx1 WT, Cplx4 WT) and mutant (Cplx1 M, Cplx4 M) peptides from retina detergent extract (Load). Beads saturated with cysteine were used as additional negative control. **(D)** Mass spectrometric quantification of the SNARE proteins Stx1, SNAP25, VAMP2, and Stx3 after affinity purification with Cplx4 WT and Cplx4 M (*n* = 3). Shown is the averaged protein abundance relative to that of SNAP25.

To gain mechanistic insight into the function of Cplx4 in regulating the size of the RRP, we searched for interaction partners of Cplx4. Because at least Cplx1 and Cplx2 were described to execute their function via binding to assembled SNARE complexes (McMahon et al., 1995; Pabst et al., 2000; Xue et al., 2007), we developed an affinity purification approach for the enrichment of SNARE complexes on the basis of synthetic peptides. In contrast to the use of fusion proteins, this allowed us to reproducibly generate efficient affinity matrices, including appropriate negative control beads with mutant peptides. We chose short Cplx fragments covering the central α-helical SNARE-binding domain, which retain the high affinity of Cplx holoproteins to fully assembled SNARE complexes (Bracher et al., 2002; Chen et al., 2002; Kümmel et al., 2011; Xu et al., 2013; Zhou et al., 2017). We synthesized such SNARE-binding peptides derived from Cplx1 and Cplx4 (Cplx1 WT, Cplx4 WT; Figure 4A), along with SNARE binding-deficient mutant peptides designed on the basis of amino acid exchanges known to abolish SNARE complex binding (Xue et al., 2007) (Cplx1 M, Cplx4 M; Figure 4A), and covalently immobilized them on agarose beads. Using these Cplx peptides as baits in combination with quantitative MS or immunoblotting, we expected to identify SNARE complex components as well as interactors of assembled SNARE complexes (Figure 4B).

To technically validate our notion that constituents of the neuronal SNARE complex can be enriched from retina homogenate by synthetic Cplx peptides, we first used affinity matrices comprising Cplx1 WT or Cplx4 WT, as well as Cplx1 M or Cplx4 M. The affinity matrices were incubated with detergent-solubilized proteins from retina homogenates, and after washing, eluted proteins were analyzed by immunoblotting. We found that the three major neuronal SNARE proteins Syntaxin 1 (Stx1), SNAP25, and VAMP2 (Synaptobrevin 2) were efficiently enriched by both Cplx1 WT and Cplx4 WT, but not by the respective mutant peptides Cplx1 M and Cplx4 M, underscoring the specificity of the enrichment. The same held true for Syntaxin 3 (Stx3), which substitutes for Stx1 in retinal ribbon synapses (Figure 4C; Morgans et al., 1996; Curtis et al., 2010). While these findings may have been expected for Cplx1 WT, derived from an established SNARE binder, the fact that the immunoblotting results with Cplx4 WT were similar in both pattern and intensity is important for two reasons: it demonstrates that our experimental design is highly suitable to capture assembled SNARE complexes by Cplx-derived synthetic peptides, and it supports the notion that the central α-helical domain mediates SNARE complex binding of all members of the Cplx protein family.

To test whether our approach can be applied in an unbiased manner for screening purposes, we performed three independent affinity purification experiments using Cplx4 WT and Cplx4 M, and subjected the eluted proteins to direct in-solution digestion, followed by quantitative MS. Abundances of the neuronal SNARE proteins and the ribbon synapse-specific Stx3 as derived from the proteomic data (Supplementary Data 2) indicated that all these proteins were enriched with Cplx4 WT, but not with Cplx4 M (Figure 4D). The observed intensity patterns and the specificity of the enrichment were confirmed by immunoblotting, demonstrating that untargeted quantitative MS can replace immunoblotting for screening purposes (Supplementary Figure 2).

### Transducin interacts with the Cplx4-mediated SNARE complex *in vitro*

**FIGURE 5:**
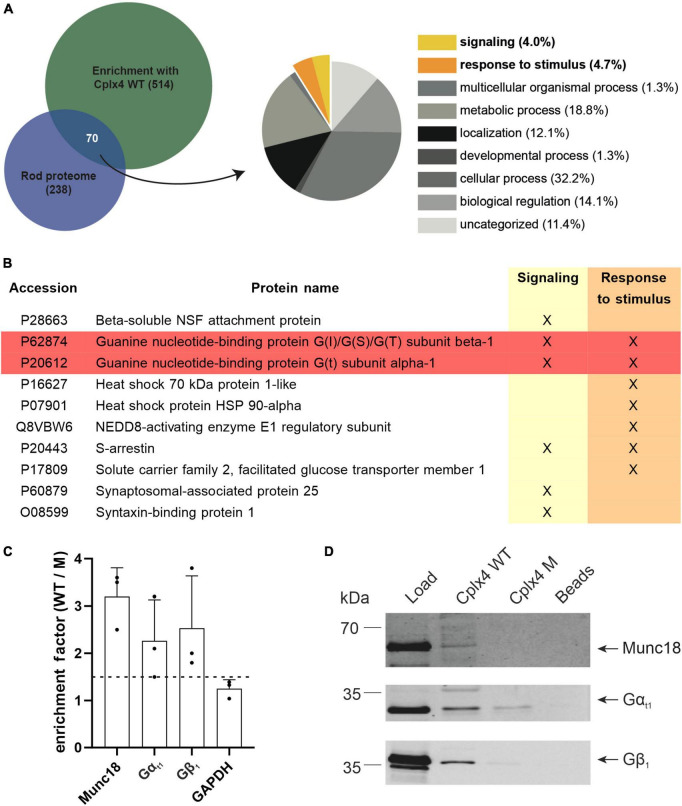
The Cplx4-SNARE complex interacts with the G protein Transducin (G_t_) *in vitro*. **(A)** Selection of candidate interactors of the rod photoreceptor Cplx4-SNARE complex. Venn diagram comparing the rod proteome (enrichment factor rod/cone >1, Supplementary Data 1, sheet 3) with the proteome enriched by the Cplx4 peptide-based affinity purification approach (enrichment factor Cplx4 WT/M > 1.5 in 2/3 experiments, Supplementary Data 2, sheet 3). The overlapping fraction was classified and the gene ontology (GO)-terms for “Biological Processes” (BP) were visualized as pie chart. The terms potentially associated with light/dark adaption are highlighted (“signaling,” yellow; “response to stimulus,” orange). **(B)** Entries classified as “signaling” (6 proteins, 4.0%) and “response to stimulus” (7 proteins, 4.7%). G_t_ subunits (highlighted in red) were selected for further study. **(C)** Mass spectrometric quantification of Munc18, Gα_t1_, Gβ_1_, and GAPDH after affinity purification with Cplx4 WT and Cplx4 M (*n* = 3). Shown is the enrichment factor as protein abundance in Cplx4 WT samples over Cplx4 M samples. Note that Gα_t1_ and Gβ_1_ showed a similar enrichment as the established SNARE complex interactor Munc18 whereas the house keeping protein GAPDH did not reach the minimal factor of 1.5 for considering a protein as enriched (dashed line). **(D)** Confirmation of the mass spectrometric protein quantification results by immunoblot detection of Munc18, Gα_t1_, and Gβ_1_.

We next used our quantitative MS approach to identify proteins that may interact with Cplx4-mediated SNARE complexes. To deduce corresponding candidates from our proteomic data, we used stringent filter criteria, only considering proteins that were quantified in all three Cplx4 WT samples and showed an enrichment factor for Cplx4 WT vs. Cplx4 M of ≥1.5 in at least two out of three experiments (Supplementary Data 2). We focused on interactor candidates (Supplementary Data 2) that were (i) rod photoreceptor specific and (ii) could potentially contribute to adaptation processes at the rod photoreceptor ribbon synapse (Figure 5A). Interesting candidates were the rod photoreceptor specific Transducin (G_t_) subunits Gα_t1_ and Gβ_1_ and Arrestin (Figures 5A, B). Since we are studying light-adapted rod photoreceptor ribbon synapses and Arrestin is localized in the OS of rod photoreceptors in light (Broekhuyse et al., 1985; Calvert et al., 2006), away from the synapse, we focused on G_t_. The subunits Gα_t1_ and Gβ_1_γ_1_ of the heterotrimeric G_t_ dissociate in bright light from the disc membranes in the rod photoreceptor OS and translocate, on a minute time scale, to the inner segment (IS) (Kassai et al., 2005). Of note, Gα_t1_ and Gβ_1_ were enriched by approximately 2.5-fold in Cplx4 WT vs. Cplx4 M samples, (Figure 5C and Supplementary Data 2), while Gγ_1_ was not identified by MS according to our stringent criteria, likely because of its small size (8.5 kDa), which leads to only a limited number of relatively small tryptic peptides. The specificity of the enrichment was confirmed by immunoblotting showing distinct intense bands of Gα_t1_ and Gβ_1_ only with Cplx4 WT (Figure 5D).

Munc18, a well-established SNARE complex interactor (Dulubova et al., 2007; Shen et al., 2007; Rodkey et al., 2008; Tareste et al., 2008; Ma et al., 2015) was chosen to validate our screening approach. It was enriched by approximately threefold with Cplx4 WT vs. Cplx4 M (Supplementary Data 2), confirmed by immunoblotting (Figure 5C). In contrast, the housekeeping protein GAPDH was not enriched (Figure 5C and Supplementary Data 2), providing further evidence that our experimental approach is suitable for the discovery of specific Cplx4-SNARE complex interactors.

### G_t_ is in close proximity to Cplx4 in light *in vivo*

In view of the interaction of G_t_ with the Cplx4-mediated SNARE complex *in vitro* (Figure 5), we next investigated a possible light-dependent spatial proximity of Cplx4 and G_t_ *in vivo*. We performed *in situ* proximity ligation assays (PLAs) (Gustafsdottir et al., 2005; Klaesson et al., 2018) on vertical cryostat sections from dark- and light-adapted WT retinae. In PLAs, oligonucleotide-tagged secondary antibodies are linked with circle-forming oligonucleotides when two antigens, detected by two primary antibodies derived from different species, are closer than 40 nm to each other. Thus, we considered a PLA signal for two proteins at the rod photoreceptor ribbon complex as an indicator for a spatial proximity of the two proteins, but not as evidence for a direct interaction. In addition to the PLA signal, application of secondary antibodies allows the visualization of the proteins of interest.

**FIGURE 6:**
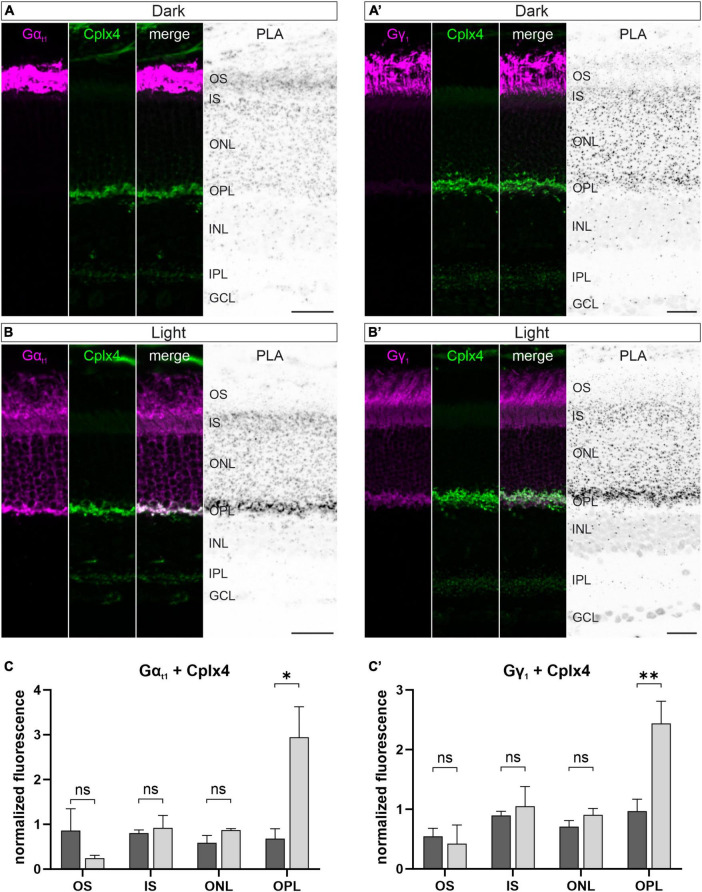
Spatial proximity of Cplx4 and G_t_ in light at rod photoreceptor ribbon synapses *in vivo*. Fluorescence micrographs of *in situ* proximity ligation assays (PLA) performed on vertical cryostat sections with antibodies against Gα_t1_ and Cplx4 **(A,B)** and Gγ_1_ and Cplx4 **(A’,B’)** after dark adaptation **(A,A’)** and light adaptation **(B,B’)**. Quantification of PLA signals in the layers of the outer retina for Gα_t1_ and Cplx4 **(C)** and Gγ_1_ and Cplx4 **(C’)**. Values are shown as mean ± SD in 3 animals. p* < 0.05, p** < 0.005, ns, not significant, *t*-test. OS, outer segments; IS, inner segments; ONL, outer nuclear layer; OPL, outer plexiform layer; INL, inner nuclear layer; IPL, inner plexiform layer; GCL, ganglion cell layer. Scale bars = 25 μm.

We first investigated the possibility of a spatial *in vivo* proximity of Gα_t1_ and Gγ_1_, in combination with Cplx4 in rod photoreceptors. We also attempted to label Gβ_1_, but corresponding antibodies did not work in our hands. This was not considered problematic because Gβ_1_ and Gγ_1_ typically form a stable dimeric complex that can only be separated under denaturing conditions (Fung, 1983). In dark-adapted retinae, Gα_t1_ and Gγ_1_ were only present in the OS and thus spatially separated from presynaptic Cplx4 in the OPL. PLA signals were distributed across the retina without any appearance of a specific labeling (Figures 6A, A’). In light-adapted conditions, both G_t_ subunits translocated from the OS across the rod photoreceptor to the synaptic terminals. Corresponding strong PLA signals in the OPL show a close proximity of Gα_t1_ and Gγ_1_ with Cplx4 in rod photoreceptor synaptic terminals (Figures 6B, B’). Quantification of PLA signal strength after light adaptation demonstrated a significant increase in the OPL (*p* = 0.02, *t*-test) for Gα_t1_ and Cplx4 (Figure 6C), and for Gγ_1_ and Cplx4 (*p* = 0.008, *t*-test) (Figure 6C’). We validated the specificity of the PLA in control experiments on vertical cryostat sections through light-adapted Cplx3/4 DKO retinae. Here, the two G_t_ subunits were translocated, but lack of Cplx4 in the rod photoreceptor terminals resulted in the absence of specific PLA signals in the OPL (Supplementary Figure 3).

In addition, we performed PLA control experiments with the G_t_ subunits Gα_t1_ and Gγ_1_ in dark- and light-adapted retinae. In dark-adapted retina, when Transducin is predominantly present in its heterotrimeric configuration Gα_t1_β_1_γ_1_, a strong PLA signal for Gα_t1_γ_1_ was detected in the OS of the rod photoreceptors (Supplementary Figure 4A). In light-adapted retina, when Transducin dissociates into the subunits Gα_t1_ and Gβ_1_γ_1_, which translocate to the IS and terminal of the rod photoreceptors, a weak PLA signal was diffusely distributed throughout the rod photoreceptors (Supplementary Figure 4B). These results further support the validity of our PLA experiments.

In summary, our PLAs demonstrate that a light-dependent association of both G_t_ and Cplx4 is possible at rod photoreceptor ribbon synapses.

### Light-induced translocation of G_t_ does not depend on Cplx4

**FIGURE 7:**
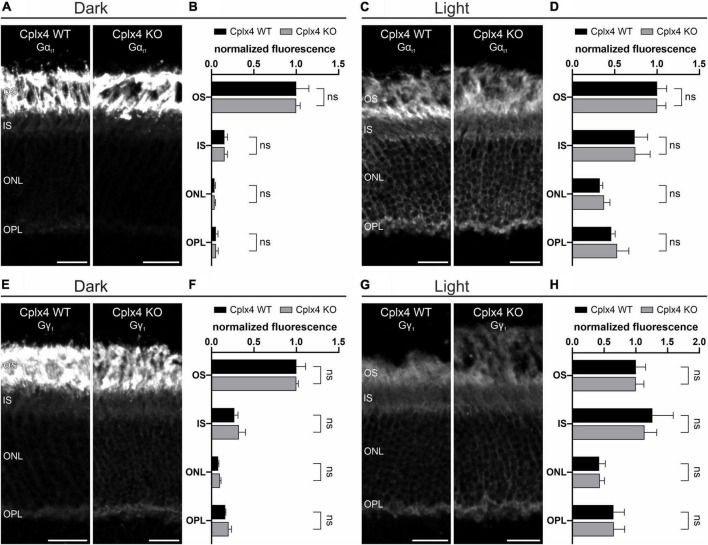
Light-induced translocation of G_t_ does not depend on Cplx4. **(A–D)** Confocal micrographs of vertical cryostat sections through Cplx4 WT and Cplx4 KO mouse retinae stained with anti-Gα_t1_ after dark adaptation **(A)** and light adaptation **(C)**. Quantification of normalized fluorescence intensity in the layers of the outer retina for Gα_t1_ after dark adaptation **(B)** and light adaptation **(D)**. **(E–H)** Confocal micrographs of vertical cryostat sections through Cplx4 WT and Cplx4 KO mouse retinae stained with anti-Gγ_1_ after dark adaptation **(E)** and light adaptation **(G)**. Quantification of normalized fluorescence intensity in the layers of the outer retina for Gγ_1_ after dark adaptation **(F)** and light adaptation **(H)**. Values are shown as mean ± SD in 4 animals; ns, not significant, *t*-test. OS, outer segments; IS, inner segments; ONL, outer nuclear layer; OPL, outer plexiform layer. Scale bars = 20 μm.

To further characterize the putative light-dependent association of G_t_ and Cplx4 at rod photoreceptor ribbon synapses, we next investigated the possibility that the translocation of G_t_ is altered in the absence of Cplx4. To this end, we examined vertical cryostat sections of dark- and light-adapted Cplx4 WT and Cplx4 KO mice labeled for Gα_t1_ and Gγ_1_. We did not find differences in Gα_t1_ staining pattern or translocation between Cplx4 WT and Cplx4 KO after dark adaptation (Figures 7A, B) and light adaptation (Figures 7C, D). The same held true for Gγ_1_ (Figures 7E–H), showing that the light-induced translocation of G_t_ does not depend on Cplx4.

### Does the association of G_t_ with the SNARE complex depend on Cplx4?

**FIGURE 8:**
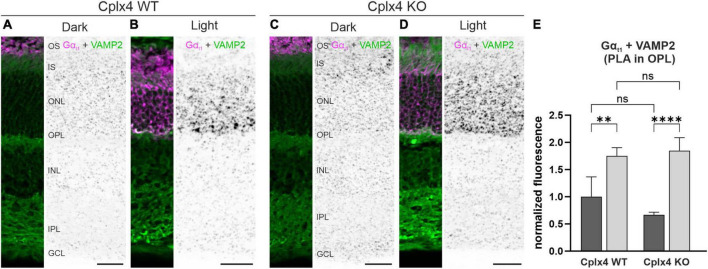
The spatial association of G_t_ and the SNARE complex at rod ribbon synapses is independent of Cplx4. Fluorescence micrographs of *in situ* proximity ligation assays (PLA) performed on vertical cryostat sections of Cplx4 WT **(A,B)** and Cplx4 KO mice **(C,D)** with antibodies against Gα_t1_ (magenta) and VAMP2 (green) after dark adaptation **(A,C)** and light adaptation **(B,D)**. **(E)** Quantification of PLA fluorescence intensity in the outer plexiform layer (OPL). Values are shown as mean ± SD in 4 animals. p** < 0.005, p**** < 0.00005, ns, not significant, ANOVA. OS, outer segments; IS, inner segments; ONL, outer nuclear layer; INL, inner nuclear layer; IPL, inner plexiform layer; GCL, ganglion cell layer. Scale bars = 25 μm.

The results of our *in vitro* (quantitative MS; Figures 4, 5) and *in vivo* (PLA; Figure 6) approaches indicate that both Gα_t1_ and Gβ_1_γ_1_ may functionally associate with Cplx4 and/or the Cplx4-SNARE complex at rod photoreceptor ribbon synapses. Having verified that the light-induced translocation of G_t_ is not altered in the absence of Cplx4 (Figure 7), we next wanted to elucidate whether Cplx4 is required for the association between G_t_ and the SNARE complex. Therefore, we performed PLA experiments on vertical cryostat sections of dark- and light-adapted Cplx4 WT and Cplx4 KO mice. We studied the subunits of G_t_ in combination with components of the rod photoreceptor ribbon synaptic SNARE complex, i.e., VAMP2, Stx3B, SNAP25. Of the available antibodies against the G_t_ subunits and the three SNARE components, only the combination Gα_t1_:VAMP2 worked reliably in our PLA experiments (Figure 8).

In dark-adapted Cplx4 WT and Cplx4 KO retinae, Gα_t1_ was only present in the OS and thus spatially separated from synaptic VAMP2 in the OPL (Figures 8A, C). Weak PLA signals were homogeneously distributed across the retina, which we interpret as background (Figures 8A, C). In light-adapted conditions, Gα_t1_ translocated from the OS across the rod photoreceptor to the synaptic terminals (Figures 8B, D). The strong PLA signals in the OPL represent the close proximity of Gα_t1_ to VAMP2 in rod photoreceptor synaptic terminals (Figures 8B, D). Quantification of PLA signal strength in the OPL after light adaptation showed a significant increase for Gα_t1_ and VAMP2 in both Cplx4 WT (*p* = 0.003, ANOVA) and Cplx4 KO (*p* < 0.0001, ANOVA) (Figure 8E). No significant differences in PLA signal strength were found between Cplx4 WT and Cplx4 KO, either after dark (*p* = 0.23, ANOVA) or after light adaptation (*p* = 0.93, ANOVA).

In summary, these results indicate a Cplx4-independent association of G_t_ with SNARE complexes at rod photoreceptor ribbon synapses in light.

## Discussion

Photoreceptor synaptic transmission is the first signal transfer step in the neuronal network of the visual system. Photoreceptor ribbon synapses can encode light signals over a wide range of intensities and maintain high SV fusion rates throughout stimulus duration. This is possible because photoreceptors can continuously and dynamically adjust their output to changes in incoming light. The SNARE complex executes Ca^2+^-triggered exocytosis of SVs at the active zone of chemical synapses (Jahn et al., 2003; Jahn and Scheller, 2006), and Cplxs are key regulators that stabilize assembled SNARE complexes and determine the speed and accuracy of excitation-secretion coupling (Reim et al., 2001; Chen et al., 2002; Wojcik and Brose, 2007; Brose, 2008; López-Murcia et al., 2019). We showed previously that Cplx3 and Cplx4 contribute to the light-dependent replenishment of SVs to the base of cone photoreceptor ribbons (Babai et al., 2016). The present study was pursued to gain mechanistic insight into the regulation of this process by Cplxs at rod photoreceptor ribbon synapses.

Since Cplx4 is the predominant Cplx isoform in rod photoreceptors (Figure 1), we examined the number of SVs tethered to rod photoreceptor synaptic ribbons by quantitative electron microscopy and the Ca^2+^-dependent sustained SV release by whole-cell patch-clamp recordings in dark- and light-adapted Cplx4 WT mice and their corresponding Cplx4 KO littermates (Figures 2, 3). At photoreceptor ribbon synapses, the first two rows of SVs at the base of the ribbon represent the RRP of SVs that mediate the fast and transient first component of SV release (Thoreson, 2021). With continued stimulation, SVs tethered higher up on the ribbon, representing the RP, replenish the RRP (Mennerick and Matthews, 1996; Datta et al., 2017; Thoreson, 2021). Our quantitative electron microscopic analysis shows that under light-adapted conditions the size of the RRP at Cplx4 WT rod photoreceptor ribbons is significantly reduced as compared to Cplx4 KO synapses, which show a similar number of SVs in the RRP as in dark-adapted conditions (Figure 2 and Table 1). The RPs were similar between Cplx4 WT and Cplx4 KO rod photoreceptors irrespective of the light conditions (Figure 2 and Table 1). Based on these results, we propose that Cplx4 is involved in a molecular mechanism that finally leads to the retardation of the SV supply to the RRP at the active zone of light-adapted WT rod photoreceptors, thereby reducing SV fusion. Our electrophysiological data are in accordance with this notion (Figure 3). Under light-adapted conditions at *V*_h_ = −40 mV, the charge transfer rate of I_AGlu_ events, representing the SV release rate, was lower in Cplx4 WT than in Cplx4 KO rod photoreceptor ribbon synapses, indicating an enhanced light signaling from rod photoreceptors to postsynaptic rod bipolar cells in WT but not in Cplx4 KO. This is consistent with our previous study, where tonic SV release was lower in WT than in Cplxs3/4 DKO cone photoreceptors (Babai et al., 2016). In principle, these data are compatible with the idea that Cplxs can clamp SV fusion in certain specialized synapses, such as ribbon-type synapses (Huntwork and Littleton, 2007; Maximov et al., 2009; Xue et al., 2010; Yang et al., 2010; Chang et al., 2015; Vaithianathan et al., 2015; Babai et al., 2016; Mortensen et al., 2016). However, in view of a key study on conditional Cplx1 KO neurons that challenges the notion of a Cplx clamp function (López-Murcia et al., 2019), and given the fact that Cplx4 KO also increases SV supply to the base of the ribbon−i.e., upstream of fusion−the increased sustained release in Cplx4 KO ribbon synapses may well be the consequence of an increased RRP size rather than a genuine unclamping of the fusion reaction.

In an attempt to understand the underlying molecular mechanism, we searched for proteins that interact with Cplx4-mediated SNARE complexes. To this end, we developed an affinity purification approach using specific peptides, representing the Cplx4 SNARE-binding region, as baits, and subsequently quantified enriched retina proteins by MS. With this unbiased approach, we identified not only all canonical neuronal SNARE complex components, i.e., the plasma membrane proteins Stx1 and SNAP25 as well as the SV protein VAMP2 (Söllner et al., 1993; McMahon and Südhof, 1995; Pabst et al., 2000; Jahn and Scheller, 2006), but also Stx3 (Figure 4 and Supplementary Figure 2), which functionally replaces Stx1 in retinal ribbon synapse SNARE complexes (Morgans et al., 1996; Curtis et al., 2010).

In addition to the SNARE complex proteins, our proteomic screen and flanking biochemical, imaging, and PLA data identified Gα_t1_ and Gβ_1_γ_1_ as interactors of Cplx4-mediated SNARE complexes *in vitro* (Figures 4, 5 and Supplementary Data 2) and *in vivo* (Figure 6). Beyond their role as key components of the phototransduction cascade in rod photoreceptor OS, the G_t_ subunits Gα_t1_ and Gβ_1_γ_1_ translocate to IS and synaptic terminals upon exposure to bright light (Brann and Cohen, 1987; Sokolov et al., 2002; Arshavsky and Burns, 2012; Srivastava et al., 2020). This G_t_ translocation is thought to reduce the phototransduction gain, thus contributing to light adaptation and reducing the metabolic stress associated with ongoing phototransduction under bright light (Sokolov et al., 2002; Majumder et al., 2013; Frederiksen et al., 2021). Furthermore, and of particular relevance to the present study, translocated G_t_ in rod photoreceptor synaptic terminals was proposed to enhance synaptic transmission to rod bipolar cells by interacting with the synaptic machinery. Although the molecular mechanism of such a G_t_-mediated synaptic control process remained enigmatic (Majumder et al., 2013), there is an interesting coincidence concerning the temporal resolution of two light-dependent processes: quantitative electron microscopy showed that in dark-adapted WT rod photoreceptors the size of the RRP decreases after about 10–15 min of light exposure (Babai et al., 2016). This time scale agrees well with the approximately 10 min that it takes G_t_ during light adaptation to translocate from the OS to the IS and synaptic terminal of mouse rod photoreceptors (Kassai et al., 2005).

Based on previous reports (Kassai et al., 2005; Majumder et al., 2013) and the results of the present study, we propose a novel presynaptic regulatory mechanism that adapts rod photoreceptor output to light intensity levels: in light, a molecular mechanism that is possibly based on a concerted action of Cplx4 and G_t_ on the SNARE complex retards SV supply to the RRP at the active zone, thereby reducing SV fusion and enhancing light signaling at the rod photoreceptor ribbon synapse as seen in electrophysiology (Figure 3).

## Conclusion and mechanistic considerations

Based on the present data, we propose that (i) the G_t_-Cplx4-SNARE association in light may minimize synaptic noise and maximize the dynamic operating range of rod photoreceptor synapses during their transition from light to dark, thus increasing contrast sensitivity and temporal resolution, and that (ii) G_t_ signals the state of light adaptation of rod photoreceptors to their synapses, where it interacts with Cplx4-regulated SNARE complexes which finally abates light-dependent SV recruitment and fusion.

Important information on how this G_t_-dependent process might operate at the protein level can be drawn from studies on the interplay between other heterotrimeric G proteins and SNARE complexes. It was suggested that Gβγ directly interacts with SNARE complexes by binding to the C-terminus of SNAP25 which prevents full SNARE zippering, interferes with the action of Synaptotagmin 1, and thereby inhibits SV fusion downstream of Ca^2+^-entry (Blackmer et al., 2001; Zhao et al., 2010; Zurawski et al., 2019). It is conceivable that a similar process operates at rod photoreceptor synapses. Although the molecular mechanism is less clear, our data suggest that Cplx4 might be involved in such a G_t_-SNARE signaling. Analogous to Cplx1 and Cplx2 in conventional synapses, Cplx4 at rod photoreceptor ribbon synapses probably stabilizes assembled SNARE complexes thereby increasing the release probability of the corresponding SVs. In the dark, this mode of action contributes to the Ca^2+^-dependent sustained release of SVs from the RRP. Upon light, G_t_ is present at the synaptic terminal, selectively binds to SNARE complexes stabilized by Cplx4 and ultimately inhibits glutamate release and/or curbs SV recruitment to the ribbon base.

## Data availability statement

The raw data supporting the conclusions of this article will be made available by the authors, without undue reservation. The mass spectrometry proteomics data have been deposited to the ProteomeXchange Consortium via the PRIDE (Perez-Riverol et al., 2022) partner repository with the dataset identifier PXD047206.

## Ethics statement

The animal study was approved by the Sachgebiet Tierschutzangelegenheiten der Friedrich-Alexander-Universität Erlangen-Nürnberg. The study was conducted in accordance with the local legislation and institutional requirements.

## Author contributions

UL: Conceptualization, Formal Analysis, Investigation, Validation, Visualization, Writing – original draft, Writing – review and editing. JM: Formal Analysis, Investigation, Validation, Visualization, Writing – review and editing. OJ: Conceptualization, Formal Analysis, Investigation, Validation, Visualization, Writing – review and editing. AD: Formal Analysis, Investigation, Validation, Visualization, Writing – review and editing. NoB: Formal Analysis, Investigation, Validation, Visualization, Writing – review and editing. AG: Conceptualization, Formal Analysis, Supervision, Validation, Writing – review and editing. AW: Formal Analysis, Investigation, Writing – review and editing. HS: Writing – review and editing. NiB: Writing – review and editing. KR: Conceptualization, Funding acquisition, Formal Analysis, Supervision, Validation, Writing – original draft, Writing – review and editing. JB: Conceptualization, Funding acquisition, Project administration, Supervision, Writing – original draft, Writing – review and editing.

## Footnotes

1
www.isoquant.net

2
www.pantherdb.org

3
https://swharden.com/LJPcalc
