# Supplementary material for: Light-dependent regulation of neurotransmitter release from rod photoreceptor ribbon synapses involves an interplay of Complexin 4 and Transducin with the SNARE complex
Source: Front Mol Neurosci. 2024 Feb 28;17:1308466. doi: 10.3389/fnmol.2024.1308466 (PMC10932955; doi:10.3389/fnmol.2024.1308466)
Supplement: Supplementary file 3 [file Data_Sheet_1.PDF]

Supplementary Material

Supplementary Data S1. Label-free quantification of cone and rod photoreceptor proteins. Related to Figure 1.

Supplementary Data S2. Label-free quantification of retinal proteins affinity-purified with Cplx4-derived peptides. Related to Figures 4 and 5.

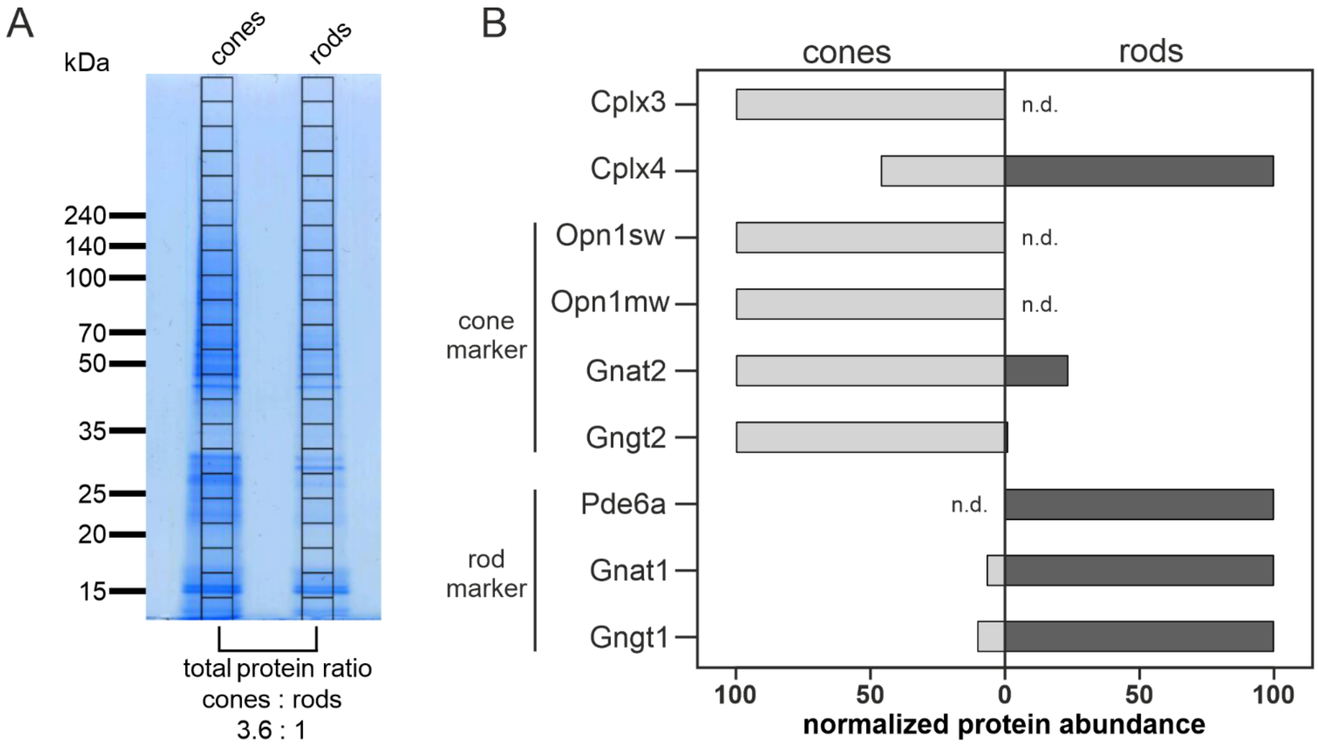

Supplementary Figure S1. Quantification of rod and cone photoreceptor proteins by a gel-based proteomic approach. **A,** Gel electrophoretic separation of proteins from identical numbers of FACS-sorted rod and cone photoreceptors (approximately 187.000 each). After protein staining with colloidal Coomassie, entire gel lanes were cut into 22 gel bands each as indicated and subjected to automated in-gel digestion with trypsin, followed by quantitative MS. For semi-quantitative comparison of selected proteins, a correction factor of 3.6 for the rod proteome was derived from the total protein as determined by quantitative MS, which was comparable to the intensity ratio of the two gel lanes as determined via Coomassie autofluorescence. **B,** Normalized protein abundance for Cplx3 and Cplx4; the cone photoreceptor markers Opn1sw, Opn1mw, Gnat2 and Gngt2; and the rod photoreceptor markers Pde6a, Gnat1 and Gngt1 in cone and rod photoreceptors as provided in Dataset S1. Bar graphs display protein abundance normalized to the maximum value detected in rod/cone photoreceptors. n.d., not detected.

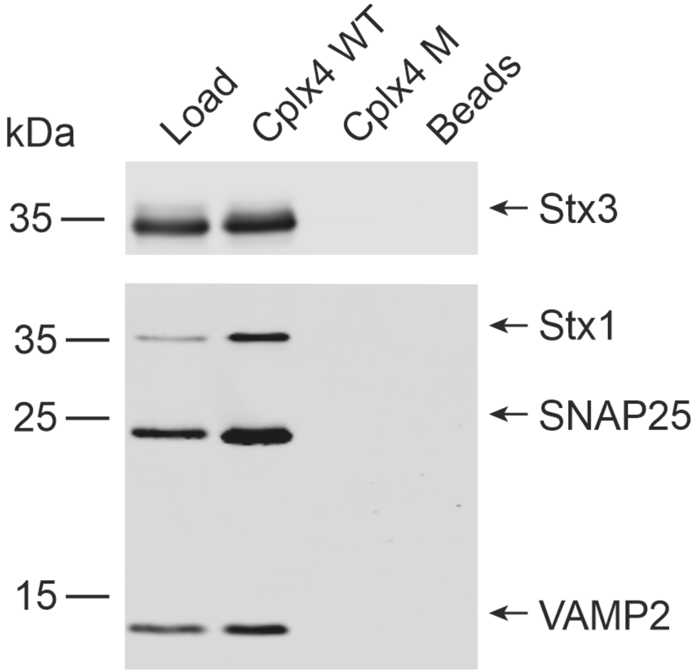

Supplementary Figure S2. Reproducibility of the affinity purification approach. Immunodetection of the SNARE proteins Stx1, SNAP25, VAMP2 and Stx3 after affinity purification with Cplx4 WT and Cplx4 M from retina detergent extract (Load). Beads saturated with cysteine were used as additional negative control. A representative immunoblot is shown from the triplicate affinity purification experiment used to screen for interaction partners of Cplx4-SNARE complexes.

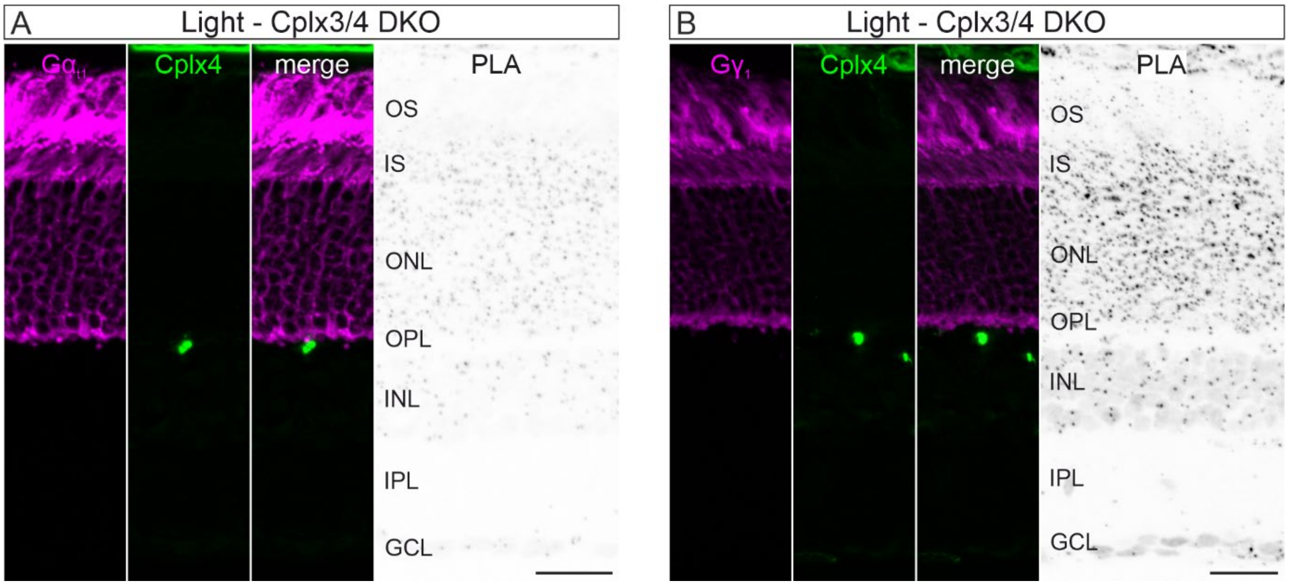

Supplementary Figure S3. *In situ* proximity ligation assays (PLA) for Cplx4 and Transducin after light adaptation in Cplx3/4 KO mice. **A-B,** Fluorescence micrographs of PLAs performed on vertical cryostat sections of light adapted Cplx3/4 KO retinae with antibodies against $G\alpha_{t1}$ and Cplx4 (**A**) and $G\gamma_1$ and Cplx4 (**B**). n = 1 animal. OS, outer segments; IS, inner segments; ONL, outer nuclear layer; OPL, outer plexiform layer; INL, inner nuclear layer; IPL, inner plexiform layer; GCL, ganglion cell layer. Scale bars = 25 µm.

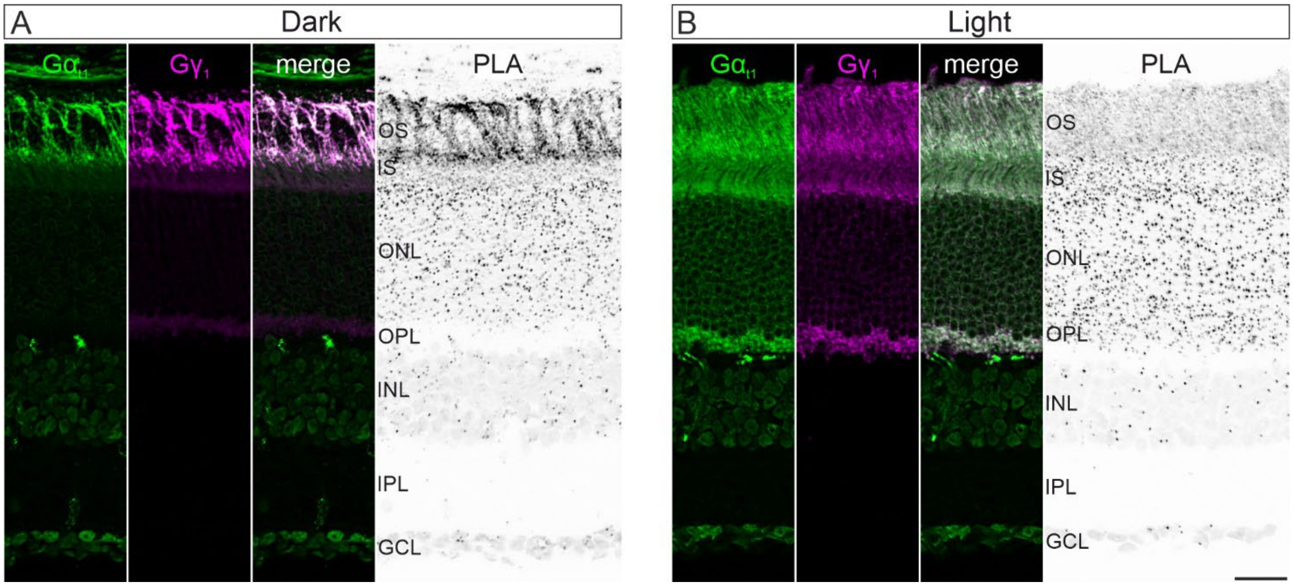

Supplementary Figure S4. *In situ* proximity ligation assays (PLA) for the Transducin subunits $G\alpha_{t1}$ and $G\gamma_1$ after dark and light adaptation. **A-B,** Fluorescence micrographs of PLAs performed on vertical cryostat sections with antibodies against $G\alpha_{t1}$ and $G\gamma_1$ after dark adaptation (**A**) and light adaptation (**B**). n = 1 animal. OS, outer segments; IS, inner segments; ONL, outer nuclear layer; OPL, outer plexiform layer; INL, inner nuclear layer; IPL, inner plexiform layer; GCL, ganglion cell layer. Scale bar in **B** for **A**,**B** = 25 μm.
